# Modeling and Inferring Cleavage Patterns in Proliferating Epithelia

**DOI:** 10.1371/journal.pcbi.1000412

**Published:** 2009-06-12

**Authors:** Ankit B. Patel, William T. Gibson, Matthew C. Gibson, Radhika Nagpal

**Affiliations:** 1 School of Engineering and Applied Science, Harvard University, Cambridge, Massachusetts, United States of America; 2 Stowers Institute for Medical Research, Kansas City, Missouri, United States of America; Stanford University School of Medicine, United States of America

## Abstract

The regulation of cleavage plane orientation is one of the key mechanisms driving epithelial morphogenesis. Still, many aspects of the relationship between local cleavage patterns and tissue-level properties remain poorly understood. Here we develop a topological model that simulates the dynamics of a 2D proliferating epithelium from generation to generation, enabling the exploration of a wide variety of biologically plausible cleavage patterns. We investigate a spectrum of models that incorporate the spatial impact of neighboring cells and the temporal influence of parent cells on the choice of cleavage plane. Our findings show that cleavage patterns generate “signature” equilibrium distributions of polygonal cell shapes. These signatures enable the inference of local cleavage parameters such as neighbor impact, maternal influence, and division symmetry from global observations of the distribution of cell shape. Applying these insights to the proliferating epithelia of five diverse organisms, we find that strong division symmetry and moderate neighbor/maternal influence are required to reproduce the predominance of hexagonal cells and low variability in cell shape seen empirically. Furthermore, we present two distinct cleavage pattern models, one stochastic and one deterministic, that can reproduce the empirical distribution of cell shapes. Although the proliferating epithelia of the five diverse organisms show a highly conserved cell shape distribution, there are multiple plausible cleavage patterns that can generate this distribution, and experimental evidence suggests that indeed plants and fruitflies use distinct division mechanisms.

## Author Summary

Cell division is one of the key mechanisms driving organismal growth and morphogenesis. Yet many aspects of the relationship between local cell division (how a cell chooses an orientation to divide) and global tissue architecture (e.g., regular versus irregular cells) remain poorly understood. We present a computational framework for studying topological networks that are created by cell division; this framework reveals how certain tissue statistics can be used to infer properties of the cell division model. Recently it has been observed that five diverse organisms show almost identical cell shape distributions in their proliferating epithelial tissues, yet how this conservation arises is not understood. Using our model we show that the low variation observed in nature requires a strong correlation between how neighboring cells divide and that although the statistics of plants and fruitflies are almost identical, it is likely that they have evolved distinct cell division methods.

## Introduction

The spatial and temporal regulation of cell shape and cell proliferation are key mechanisms that direct tissue morphogenesis during development. Much of our knowledge of tissue morphogenesis comes from the study of simple epithelial monolayers, 2D planar sheets of strongly adhering cells in which division occurs in the plane of the epithelium. The strong structural constraints and developmental importance of epithelia have inspired a multitude of theoretical and computational models since the early 20^th^ century [1]–[6]. Of these, an important class is topological models, where an epithelium is represented as a planar network (topology).

**Figure 1:**
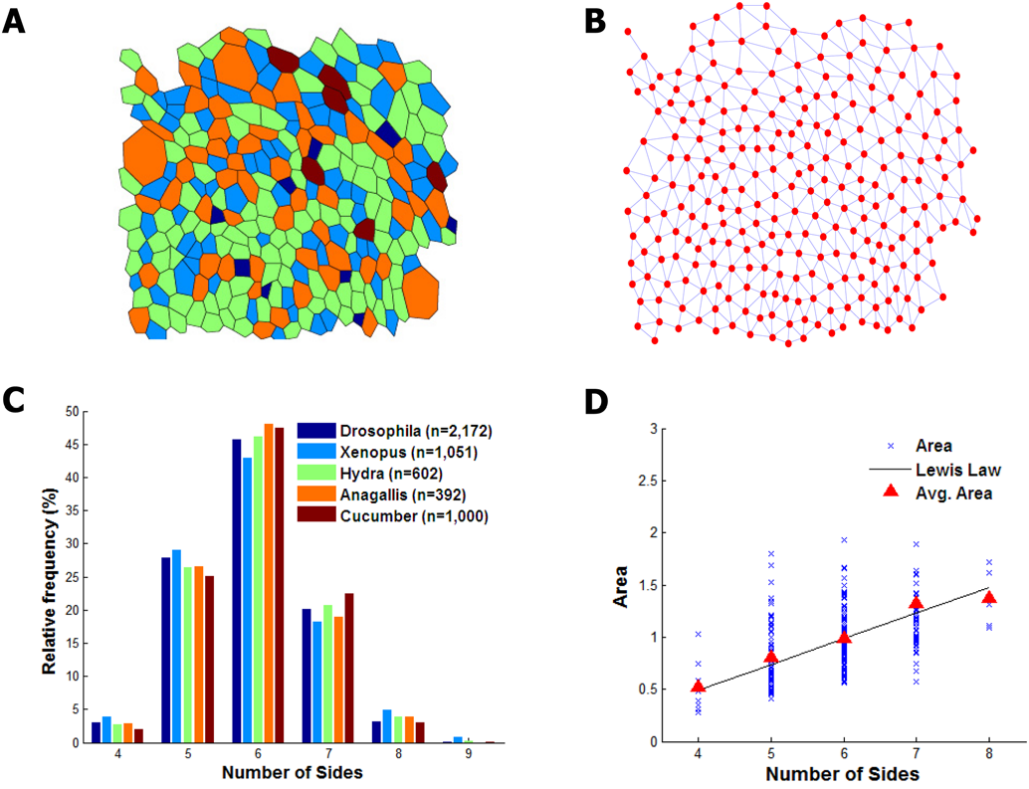
Topological properties of natural epithelia. (A) Polygonal lattice approximation of a larval stage wing disc epithelium from *Drosophila melanogaster*. Color encodes polygonal shape i.e. the number of neighbors. [darkblue = 4, blue = 5, green = 6, orange = 7, maroon = 8] (B) Underlying topology of cell-cell connections in (A); each node represents the center of a cell and edges denote cell-cell adjacency. (C) Distribution of polygonal cell shapes from the epithelia of five disparate organisms: *Drosophila melanogaster (third instar larval wing)*, *Xenopus laevis (tadpole tail epidermis)*, *Hydra vulgaris (outer epidermis)*, *Anagallis arvensis (meristem)*, cucumber epidermis [2],[5],[16]. Number of cells per sample is indicated in the legend. (D) Correlation between a cell's polygonal shape and its area in the larval *Drosophila* wing disc (2,172 cells). Cell area as a fraction of total area is shown in blue; the average area of an *n*-sided cell, *A_n_*, is shown in red. The solid line shows the expected prediction of Lewis' Law [4], *A_n_ = A_avg_ (n−2)/4*, with the average area per cell *A_avg_* = 1 without loss of generality.

The topology of an epithelium is defined as the network of connectivity between cells (Figure 1A and 1B). Some important topological properties include a cell's polygonal shape, defined as its number of neighbors, and the overall distribution of cell shapes within an epithelium. There are several reasons for considering these properties. First, empirical evidence from our recent work [5] shows that the distribution of cell shapes is conserved in the proliferating epithelia of several diverse organisms, including the *Drosophila* larval wing disc and the *Xenopus* tadpole tail epidermis (Figure 1C and Table S1). Second, polygonal cell shape is linearly correlated with cell surface area (Figure 1D), a longstanding empirical observation known as Lewis' Law [2],[3]. Third, important developmental processes such as cell division, migration, and intercalation fundamentally alter topology by creating and breaking connections between cells.

For these reasons, topological models have been useful both experimentally and theoretically in understanding proliferating epithelia [4]–[8] and other non-biological lattices [9],[10]. As early as the 1920s, F.T. Lewis documented the connection between cell proliferation and tissue topology, arguing that spatial control of cell divisions could affect the overall distribution of polygonal cell shapes [2],[3]. Since that time, the relationships between cell shape, proliferation and epithelial topology have been further investigated using both topological models [4],[5],[9],[10] and mechanical models [11]–[13], exploring a wide variety of phenomena including differential rates of division, adhesion forces, and stochastic divisions. However, due to unknown parameters and simplifying approximations, the specific mechanisms by which global tissue morphology emerges from the local control of cell divisions in epithelial monolayers still remains poorly understood. To better understand proliferation within the larval wing disc of *Drosophila melanogaster*, we recently developed a stochastic topological model of cell division [5]. Our model mathematically predicts the emergence of a specific equilibrium distribution of polygonal cell shapes (p*), revealing how local stochastic cellular processes can give rise to predictable global tissue properties.

The predicted distribution p* was empirically confirmed in the larval imaginal wing disc of *Drosophila melanogaster*, but also closely matched in the tadpole tail epidermis of *Xenopus laevis*, the outer epidermis of the cnidarian *Hydra vulgaris*, and also Lewis's cucumber epidermis (Figure 1C). A common characteristic across these diverse examples is that the epithelia-like tissue undergoes rapid proliferation with minimal cell rearrangement. The apparent conservation of p* across these systems is surprising. Is p* the consequence of a conserved process of cell division across these proliferating 2D epithelia? Or is it possible that distinct processes of cell division converge upon the same final distribution of shapes? More generally, how do widely varying division strategies impact global epithelial organization?

Despite much experimental and theoretical progress, previous models have limitations that make it difficult to address these questions. The major difficulty lies in modeling the neighborhood and lineage dependence in cleavage plane choice. For example, our previous model encodes a mean-field approximation that ignores the variability in the number of neighbors gained via the division of neighboring cells [5]. The mathematical models of Cowan et al. [9] do not account for neighboring divisions at all: a cell never gains sides from its dividing neighbors. These models cannot be used to study modes of cell division with any spatial or temporal dependence, both of which are biologically relevant. For example, cleavage patterns with mother-daughter or neighbor-neighbor correlations in cleavage plane choice are common [14]. To explore and characterize the space of plausible cleavage patterns, a more expressive model is required.

Here we present a computational model of cell division that enables us to explore a much larger class of biologically plausible division models by directly simulating the topology of a proliferating epithelium from generation to generation. This includes division schemes with spatial and temporal dependence between neighboring cells and mother-daughter cells. Given a division model, we can compute the equilibrium distribution of polygonal cell shapes, along with other tissue-level topological properties. Our findings show that the fraction of hexagons and the variability in cell shape are both important global indicators of local division parameters, and we propose that it may be possible to infer these parameters from empirical data. Furthermore, we describe several division schemes that can reproduce with high accuracy the cell shape distribution seen in five diverse organisms. We use this modeling framework to formulate and explore some of the central theoretical and empirical questions regarding the local-to-global regulation of cell shape in proliferating epithelia.

## Model

The topology of an epithelial cell sheet can be described mathematically as a planar network of trivalent vertices, edges, and faces. The vertices represent tricellular junctions, the edges represent cell sides, and the faces represent the cells themselves (Figure 1A). This planar network captures the connectivity between cells, but ignores geometric properties such as area, perimeter or interior angles. In this paper, we are interested in a cell's topological shape, which is defined as its number of sides, or equivalently, its number of neighbors in the planar network. Cell division events within the network locally alter the topology of the planar graph by adding new vertices, edges, and faces; multiple rounds of proliferation can thus significantly alter global tissue topology. By representing cell proliferation as a computation on an epithelial network, one can simulate many different cell division strategies and study the emergence of global properties such as the distribution of topological cell shape.

**Figure 2:**
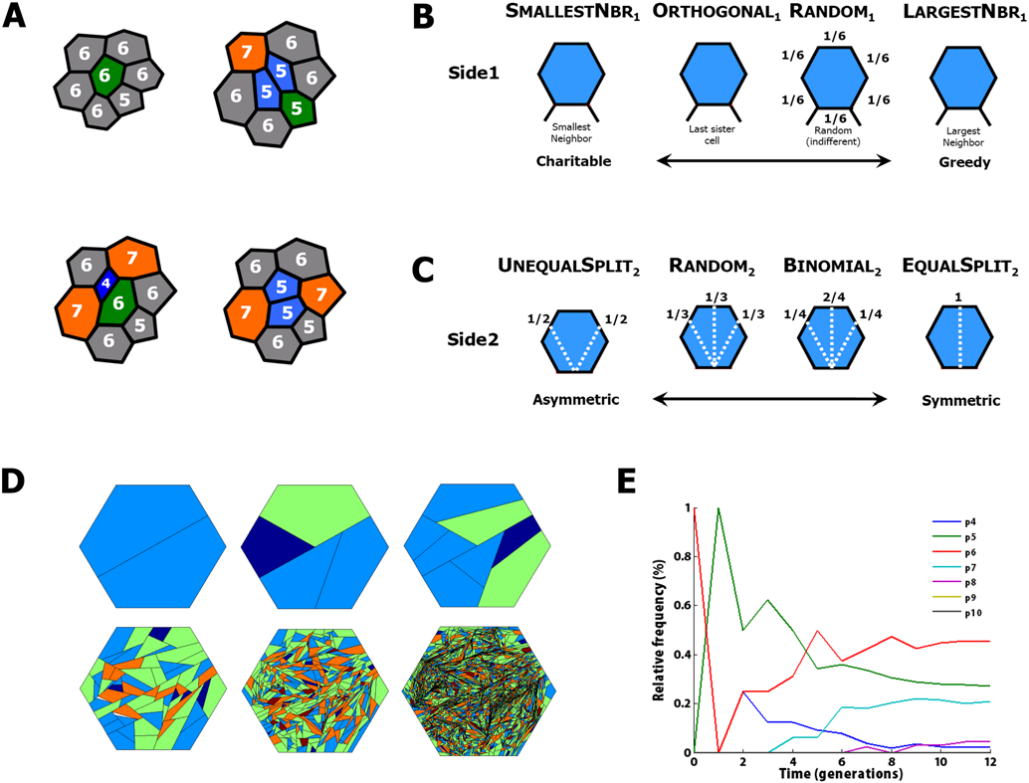
Simulating cleavage plane models. (A) A cell's cleavage plane model (CPM) specifies the stochastic rule by which a cell chooses its cleavage plane for the next division. In this example, the hexagonal mother cell has equal chance of dividing into two pentagons or one hexagonal and one quadrilateral cell. The choice of cleavage orientation can also affect the neighbor cells in more than one way, for example it may be biased towards smaller neighboring cells. After division, daughter cells lose sides on average, while two neighboring cells gain sides (orange). (B,C) A CPM is specified by the choice of first edge (B) and second edge (C). The possible cleavage planes are shown as dashed white lines. Probabilities of choosing a cleavage plane are shown adjacent to the second edge. (D,E) Dynamics of the Orthogonal|EqualSplit CPM for 12 generations for an initial condition of one hexagonal cell. (D) Generations *t* = 1,2,3,6,9,12 are shown. Color encodes polygonal shape. Note that the diagram represents topological connectivity between cells and does not model areas, angles, and perimeters of cells. (E) Shape distribution for Orthogonal|EqualSplit CPM for all 12 generations of a single run.

The core of the topological model is the *cleavage plane regulation model* (CPM), which describes how a cell determines which two of its sides will be bisected by the cleavage plane (Figure 2A–C). Based on experimental observations of the *Drosophila* larval wing disc and other proliferating epithelia [5], we define the set of assumptions that underlie our proliferation model.

### Model Assumptions

- The epithelial network is only modified by cell division. We do not consider any junctional rearrangements due to cell repacking, cell migration, or cell death.
- Each cell divides exactly once per division cycle and the order in which cells divide is chosen uniformly at random from all possible orderings. All cells in an epithelium use the same algorithm, or CPM, for choosing their cleavage plane.
- A parent cell divides into two daughter cells through the creation of two trivalent vertices and one edge along the chosen cleavage plane. Thus daughter cells always share an edge (Figure 2A).
- When a cell divides, its cleavage plane must consist of two non-adjacent edges of the original cell. This precludes the formation of tetravalent vertices and 3-sided cells, both of which are rarely observed empirically.

This model describes a generic proliferating epithelium with no/minimal cell rearrangement. The assumptions are based on experimental evidence from the larval stage wing disc of *Drosophila melanogaster*, where the absence of cell rearrangement, roughly uniform cell division rates, and cleavage plane restrictions, appear to hold [5]. These assumptions also appear to be approximately valid for the other proliferating epithelia presented in Figure 1, for example in plants, where rearrangement does not occur [2],[3]. However in some cases, rearrangement may occur more frequently and there may be a higher occurrence of tetravalent vertices and three-sided cells; for those systems the model can be modified to include those aspects, although this is beyond the scope of the current paper.

#### Cleavage Plane Regulation Model (CPM)

The CPM is the core of the model and describes how mitotic cells select their cleavage planes. The two main local parameters of a CPM that affect global epithelial topology are: 1) The extent to which cleavage plane orientation is directed by the local neighborhood surrounding the cell; and 2) The symmetry with which a mitotic cell's neighbors are distributed to the two daughter cells. Computationally, this is modeled as a two-stage algorithm that first selects a cell side (Side1) based on local topology and then selects a second side (Side2) based on topological symmetry (Figure 2B and 2C). In the final step, cell division occurs through creation of a new side connecting Side1 and Side2.

The selection of Side1 models the influence of local neighborhood topology on cleavage plane orientation. Biologically, the local topology surrounding the cell could impact cleavage orientation if neighbors with fewer sides influence physical properties such as tension in the mitotic cell cortex [12],[13]. The cleavage plane could also be influenced by a historical correlation between the mother and daughter cleavage plane orientations [14],[15]. To model these effects, we simulated four strategies for the choice of Side1: Random_1_, Smallest Neighbor_1_, Largest Neighbor_1_ and Orthogonal_1_ (Figure 2B). The four Side1 strategies are described below (for equations see Text S1):

- **Random_1_.** A critical default scenario for cleavage plane orientation is that alignment of the mitotic spindle proceeds without regard to local epithelial topology. To model this situation, Side1 is chosen uniformly at random from all cell sides. This strategy mimics a geometric model where neighbor cells play no significant role in cleavage plane choice.
- **Smallest Neighbor_1_.** A second conceivable mechanism for cleavage plane orientation is that the mitotic spindle apparatus senses local topology and aligns such that the smallest neighbor will gain a side in the subsequent division. To model this situation, Side 1 is chosen to be the neighbor with the smallest number of neighbors. This strategy topologically mimics the case where the smallest neighbor exerts the most tension on the cell and the cleavage plane attempts to relieve some of that tension by dividing in its direction.
- **Largest Neighbor_1_.** Again we assume that the local topology is sensed by the dividing cell. However, in contrast to the Smallest Neighbor_1_ model, here Side1 is chosen from the neighboring cell with the largest number of neighbors. Though biologically implausible, it will help us assess the impact of division asymmetry on global tissue topology.
- **Orthogonal_1_.** This CPM mimics orthogonal regulation, a strategy known to be common in plants [14],[15], where the cleavage plane rotates by 90° with each successive cell division. Topologically, the side that a cell shares with its sister cell from the previous division will be chosen as Side1.

A second important factor in the determination of cleavage plane orientation is the manner in which a mitotic cell's neighbors are segregated between the two daughter cells. We refer to cell divisions that equally segregate neighbors as symmetric, while divisions that segregate neighbors unequally are asymmetric. The symmetry of a CPM is governed by the choice of Side2, the second edge of the cleavage plane. We simulated four strategies for the selection of Side2: Random_2_, EqualSplit_2_, Binomial_2_, and UnequalSplit_2_, all of which are illustrated in Figure 2C. The four Side2 strategies are described below (for equations see Text S1):

- **Random_2_** (indifferent). Side2 is chosen uniformly at random from all edges not adjacent to Side1. Under this strategy, symmetric cleavage planes are as equally likely to be chosen as asymmetric ones.
- **EqualSplit_2_** (maximally symmetric). Side2 is chosen so as to divide a mitotic cell's tricellular junctions as equally as possible amongst its two daughters. This strategy mimics a typical geometric model where cell junctions are (roughly) evenly spaced around the cell and cleavage planes are diameters that cut the cell into two daughters of approximately equal area.
- **Binomial_2_** (moderately symmetric). Side2 is chosen according to a binomial distribution from all edges not adjacent to Side1. In this strategy, symmetric outcomes are more probable than asymmetric ones. The geometric equivalent of this topological strategy assumes that junctions are placed uniformly at random around the cell periphery. Thus each cell junction has equal chance of belonging to either daughter upon division provided the cleavage plane does not produce 3-sided cells. This strategy was modeled mathematically in [5].
- **UnequalSplit_2_** (maximally asymmetric). Side2 is chosen such that the cleavage plane divides a cell as unequally as possible. Under this strategy, an *n*-sided cell will produce one 4-sided daughter and one *n*-sided daughter after division. This biologically implausible strategy tests the impact of severely asymmetric divisions.

#### Simulation methodology

**Figure 3:**
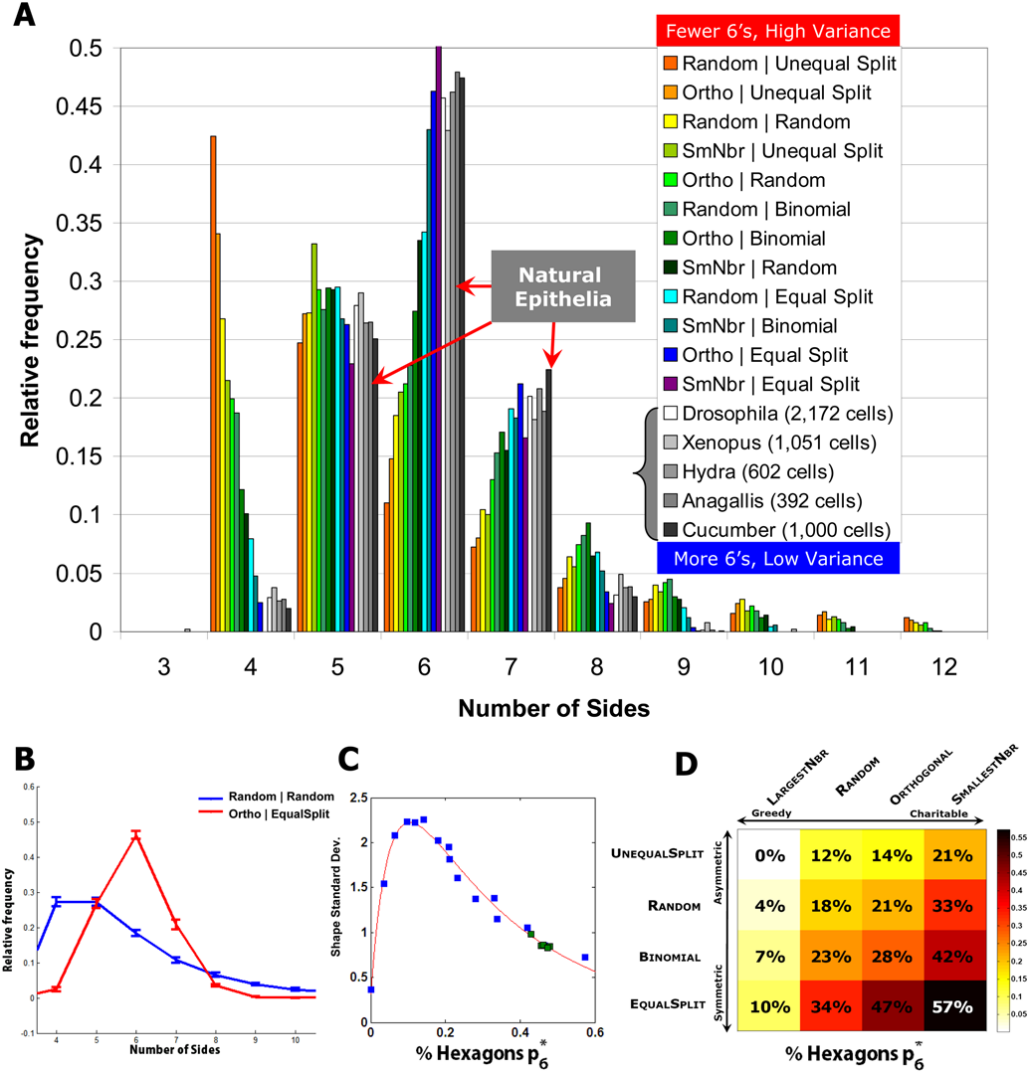
Convergence of proliferating epithelia to an equilibrium distribution. (A) Steady-state shape distributions for all simulated CPMs (color), sorted from high to low cell shape variance. Also included are the proliferating epithelia (grayscale) from Figure 1; these epithelia have lower variance than all but one simulated CPM (SmallestNeighbor|EqualSplit). (B) Equilibrium cell shape distributions for the stochastic Random|Random CPM and the deterministic Orthogonal|EqualSplit CPM with an initial condition of a single cell with *S_0_* sides, where *S_0_* ranges from 4 to 250 sides. Probabilities are mean over all runs and error bars represent range. (C) In the simulated CPMs, high hexagonal frequency is strongly correlated with lower cell shape variability as measured by standard deviation. Proliferating epithelia data (green) shows a similar relationship between high hexagonal frequency and low shape variability. (D) The fraction of hexagons in the equilibrium shape distribution for all simulated CPMs. Rows and columns correspond to the choice of first and second edge, respectively, and colors encode the resulting fraction of hexagons after generation 12.

Each pair of Side_1_ and Side_2_ algorithms constitutes a distinct CPM, denoted by Side1|Side2. We simulated each of the 16 possible CPMs for a total of 12 generations of cell division. In each generation, every cell divides once and the order in which cells divide is random. We simulated many different initial conditions (a single *m*-sided polygon for 3<m<250). For each initial condition, the simulation was run 100 times. All simulations yielded 2^12^ = 4,096 cells. For each simulation, we recorded the final topological shape distribution and the CPM mean and standard deviation over 100 trials (Figure 3A and Table S2, S3, and S4). As an example, results from the simulation of the Orthogonal|EqualSplit CPM are shown in Figure 2D and 2E. Simulation models were implemented in Java and data analysis was done using MATLAB and Microsoft Excel.

## Results/Discussion

### Characterizing the Space of CPMs

#### All CPMs generate an equilibrium cell shape distribution

Previous work suggests that proliferating epithelia with a specific CPM will converge to an equilibrium distribution of polygonal cell shapes [4],[5]. Whether this holds true for every possible (side_1_|side_2_) CPM remains an important question. We find that simulations of a completely random CPM (Random|Random) and a completely deterministic CPM (Orthogonal|EqualSplit) both converged to distinct equilibrium distributions independent of initial conditions; the standard deviation in the percentage of hexagons was less than 0.5% for both CPMs over widely varying initial conditions (Figure 3B and Table S2, S3, and S4). Similar results were obtained with the 14 other CPMs (Table S4). Together, these findings indicate that all CPMs converge to a predictable, fixed distribution of polygonal cell shapes independent of the initial topology. The intuition is that the initial conditions become statistically insignificant as the number of cells expands exponentially through division.

#### Frequency of hexagons and overall cell shape variability characterize cleavage patterns

Assuming negligible boundary effects, every CPM described herein should converge at an exponential rate to a mean shape of 6 sides [2],[3],[5] (Text S1). Thus, the mean cell shape in equilibrium cannot distinguish between CPMs (Figure S1). In contrast, CPMs are strongly distinguished by their equilibrium cell shape variance and also by the percentage of hexagons and quadrilaterals in the population (Figure 3C and Table S2 and S3). These statistics vary significantly from CPM to CPM and are strongly correlated with two key properties of the CPM: neighbor *charitability* and division *symmetry*. Each division event splits one mitotic cell into two daughters with fewer neighbors on average; simultaneously it increases the number of sides for two neighbors of the mitotic cell. *Charitability* refers to the tendency of the Side1 choice to confer sides to smaller neighbors, potentially reducing cell shape variation within the local neighborhood (Figure 2A, upper right and Figure 2B, Smallest Neighbor_1_). *Symmetry* refers to the tendency of the Side2 algorithm to create two daughter cells with equal numbers of neighboring cells (e.g., Figure 2C, EqualSplit_2_). Our findings indicate that highly symmetric and charitable CPMs suppress global cell shape variability.

#### Symmetric, charitable cleavage patterns amplify percentage of hexagons and suppress variation in cell shape

Our simulation results reveal a strong correlation between the degree of division symmetry and the number of hexagons in the population. For every Side1 strategy tested, the percentage of hexagons in the population increased with increasingly symmetric Side2 CPMs (Figure 3D). This increase in hexagons was accompanied by a substantial decrease in the variance (Figure 3A and 3C). Consistent with this result, strongly asymmetric CPMs yielded an equilibrium distribution with a mode of 4 or 5 sides, suggesting that symmetric divisions may be critical to establishing the majority of hexagonal cells observed in most natural epithelia.

The degree with which the Side1 CPM favors smaller neighbors also had a noteworthy effect on the percentage of hexagons in the population. One can order LargestNeighbor_1_, Random_1_, SmallestNeighbor_1_ as explicitly increasing in charitability. For every Side2 algorithm tested, increasingly charitable Side1 CPMs led to an increased percentage of hexagons and a correspondingly lower variance (Figure 3A, 3C, and 3D and Table S2 and S3). Orthogonal_1_ appears to be implicitly charitable; the CPM favors the recently divided sister cell which tends to have fewer sides due to its recent division. The simulations suggest that this CPM lies between Random_1_ and SmallestNeighbor_1_ in its ability to reduce shape variance. The simulations also reveal some complexities overlooked by our earlier Markov chain model [5], which assumes binomial symmetry but approximates the effect of neighbor correlations using a mean-field assumption. The simulations show that many cell shape distributions are possible, given a binomial division symmetry model. In order to produce a fraction of hexagons close to that observed in natural epithelia (>40%), the Side1 model must have high charitability (e.g., SmallestNeighbor_1_|Binomial_2_). Also, a different CPM (Orthogonal_1_|EqualSplit_2_) can reproduce the cell shape distribution observed in natural epithelia; this CPM has lower charitability but higher symmetry. This illustrates that the interplay between autonomous symmetry and non-autonomous charitability critically determine the equilibrium shape distribution.

#### Minimum and Maximum Variance Cleavage Patterns

The CPM that minimized the variance in polygonal cell shape and produced the largest percentage of hexagons was SmallestNbr_1_|EqualSplit_2_, which is both maximally charitable and maximally symmetric (*p_6_* = 58%, σ = 0.73 sides, *p_4_* = 0%). At the other end of the spectrum is LargestNbr|UnequalSplit, a biologically implausible strategy that is maximally uncharitable and maximally asymmetric, and which generates a highly distorted topology dominated by quadrilaterals (*p_6_* = 0.2%, σ = 3.41 sides, *p_4_* = 97.2%, see Table S2, S3). These CPMs represent the extremes for the symmetry and charitability parameters. Many existing proliferation models [4],[5],[12] produce distributions within this spectrum, and our results provide insights into the distributions generated by mechanical models [12] as well as the distributional shift observed in mitotic cells ([2]–[4] and see Text S1 and Table S5 for comparisons to other relevant models). Notably, we were unable to find a CPM that generates more than 60% hexagonal cells, suggesting that it may be difficult to achieve higher hexagonal fractions with solely local information. Indeed, natural epithelia with higher regularity (80% hexagonal) appear to involve mechanisms with significant cellular rearrangement and global signaling [8].

### Comparison to Empirical Data

The wide spectrum of shape distributions produced by different CPMs raises the intriguing possibility of inferring the CPM based solely on empirical observations of global epithelial topology. For example, a hypothesis for a cell division strategy in a given epithelium might be rejected simply by comparing the empirical cell shape distribution with the one predicted by the CPM. Here we present the results of comparing our simulated CPMs to cell shape distribution data from natural proliferating epithelia in a diverse array of organisms. We use data, collected and published previously by our group [5], on *Drosophila melanogaster* (larval wing disc, arthropod), *Xenopus laevis* (tadpole tail epidermis, vertebrate), and *Hydra* (adult outer epidermis, cnidarian). In addition, we have included previously published data from two plants, *Cucumis* (cucumber epidermis) from the paper by F.T. Lewis [3] and *Anagallis arvensis* (meristem) courtesy of J. Dumais [16]. These natural epithelia show a strongly conserved cell shape distribution with between 42–48% hexagons and a standard deviation of 0.83–0.98 sides (Figure 1C and Table S1).

#### Natural epithelia exhibit relatively low variation in cell shape

To compare simulated CPMs with natural epithelia, we sorted all distributions (simulated and empirical) by variance. Compared with simulated topologies, natural distributions exhibit a surprisingly low shape variance and a high percentage of hexagons (Figure 3A and 3C). Only the SmallestNbr|EqualSplit CPM had a lower variance (σ = 0.73 sides). It is unclear why natural epithelia should exhibit such low variance in cell shape. One conjecture is that if cell size (area) is proportional to cell shape (number of sides), then low variability in cell shape is consistent with low variability in cell size. To test this hypothesis, we measured the correlation between a cell's number of sides (*n*) and its geometric area in the *Drosophila* wing disc. Our results, shown in Figure 1C, show a linear correlation between *n* and *A_n_*, the average size of an *n*-sided cell, consistent with Lewis' Law [2],[3]. However, there is significant variability in cell size about the mean *A_n_*. Alternatively, the low shape variability may be an indirect outcome of other factors that favor specific division mechanisms.

#### Two distinct CPMs generate the distribution observed in natural epithelia

Of all division models tested, the Orthogonal|EqualSplit CPM most closely matched the empirical natural cell shape distribution data (Figure 3A and 3B and Table S1). Surprisingly, this CPM is deterministic: cells choose cleavage planes based solely on the location of the last sister cell (Figure 2B and 2D). It yields 46% hexagons, and a standard deviation of σ = 0.84 sides, similar to *Drosophila* and *Cucumis* [2],[5]. Consistent with natural epithelia, it also generates a negligible fraction of cells with 10 sides or greater (<1 in 10^4^) and has a nonzero fraction of 4-sided cells (*p_4_* = 2.4%), close to the empirically observed frequency of 2.95% in *Drosophila*. This significantly improves upon the prediction of our Markov chain model [5], where the mean-field approximation incorrectly yields *p_4_* = 0%. To a lesser extent, the SmallestNbr_1_|Binomial_2_ CPM also matches the empirical data, with 43% hexagons and a standard deviation of 0.72 sides and 5.3% 4-sided cells. Although this is significantly different from the conserved empirical distribution, it is possible that a similar CPM with higher symmetry than Binomial_2_ but lower symmetry than EqualSplit_2_ may generate the expected distribution. We have derived such a CPM through simulation (Figure S2).

#### Is the conserved natural distribution due to a conserved division strategy?

Previous results raise the possibility that the conserved distribution may arise from distinct division strategies in different organisms. To test this possibility, we compare our simulated distributions to those found in related work on cell division in plants and in the larval wing disc of *Drosophila melanogaster*.

#### Cell division in plants

Orthogonal regulation is a common mode of division in plant development [1],[2],[15],[16]. For example, spindles in some plant cells use microtubules to find the longest axis and divide perpendicular to that axis [2], [15]–[18]. This corresponds topologically to the CPM Orthogonal|EqualSplit, provided that cell growth is isotropic and daughter cells are roughly equal in size, as is the case in the *Cucumis* epidermis and the central region of the *Anagallis* meristem. To illustrate, consider a rectangular cell with width greater than its height. Division along the short vertical axis will yield two rectangular cells of height greater than width; thus the next cleavage plane will be in the horizontal direction, perpendicular to the parent's cleavage plane [18]. Since the next cleavage plane usually emanates from the newly created cell wall, this is consistent with the Orthogonal_1_ rule. Thus, the Orthogonal|EqualSplit CPM is a good topological approximation to the original geometric rule in some plants.

#### Cell division in the Drosophila wing disc

Although the *Drosophila* wing disc has a shape distribution almost identical to that of the plants *Anagallis* and *Cucumis*, there is significant evidence to suggest that orthogonal regulation does not occur in the fly. Specifically, it is known that the orientation of the first cell division is often maintained in subsequent divisions, with 57% of four-cell clones forming a straight line of one cell width [19]. Also, most clones in the wing blade are elongated and grow along the proximal-distal axis, perpendicular to the dorsal-ventral border [20]. This type of region-specific oriented division rules out purely orthogonal regulation, where four-cell clones should form 2×2 diamonds. In addition, orthogonal regulation predicts roughly circular clone shapes.

Given the evidence against Orthogonal|EqualSplit in the fruitfly, our simulations suggest trying a maximally charitable and moderately symmetric CPM that lies somewhere between SmallestNeighbor|EqualSplit and SmallestNeighbor|Binomial. To test this idea, we interpolate between the two CPMs using a parameter 0<*a*<1, and we find that a good fit to the empirical shape distribution is achieved at *a* = 0.75 (Figure S2). However, it is unclear how the SmallestNeighbor_1_ might translate into a physical mechanism. One possibility is that for a given cell, the longest edge is adjacent to the smallest neighbor and thus more likely to be cut by a cleavage plane or exert the most tension [13]. Alternatively, favorable neighbor correlations might arise indirectly, as a result of globally aligned divisions [19]. Nevertheless, it is clear that some form of charitability is required. A recent mechanical model of cell division in the wing [12] uses data-derived parameters to replicate cell geometry but assumes that the division orientation is unaffected by cell neighbors. Our topological model predicts that such a system, with moderate symmetry but indifferent to local neighborhood, is likely to have more 5-sided cells than 6-sided cells, as observed in the mechanical model. Understanding how charitability arises will require a more thorough investigation of the division parameters in the *Drosophila* wing, which are still poorly understood.

By comparing natural and simulated cell shape distributions, we can make several inferences about proliferating epithelia. First, the observed low variability in cell shape implies that division strategies are not only highly symmetric, but also moderately charitable: they directly or indirectly favor adding sides to smaller neighbors. Second, although the proliferating epithelia of five diverse organisms show a highly conserved shape distribution, there are multiple plausible CPMs that can generate this distribution, and experimental evidence suggests that indeed plants and fruitflies do have distinct division mechanisms. This raises the possibility that different organisms may have evolved distinct mechanisms to suppress shape variability during proliferation. Alternatively, the low shape variability may be an indirect outcome of other factors that favor symmetric and charitable divisions. Looking forward, as proliferation is better understood in other organisms, our topological framework can provide a background for hypothesis generation and testing as well as a basis for studying pattern formation in the presence of proliferation.

## Supporting Information

Figure S1 Hexagonal fraction p6* vs. mean shape. Most CPMs produce a mean shape close to 6, even though the percentage of hexagons varies significantly. A mean of 6 is expected for all CPMs, provided that the boundary effects are minimal. Only a few CPMs, based on LargestNeighbor1 show a mean closer to 5, as discussed in the Supplementary text.
(3.46 MB TIF)

Figure S2 A SmallestNeighbor based CPM that matches empirical data. We interpolate the symmetry value between SmallestNeighbor|Binomial and SmallestNeighbor|EqualSplit by having each cell execute the first method with probability a and the second method with probability (1-*a*). Thus, a measures distance from maximally symmetric to moderately symmetric division. Best fit to empirical distribution (light and dark green) and to the alternative CPM (Orthogonal|EqualSplit) is achieved by *a* = 0.75 (blue).
(5.22 MB TIF)

Table S1 Empirical Cell Shape Distribution Data from Five Diverse Organisms. Shape distribution data for proliferating epithelial in several organisms of interest. The data for *Drosophila melanogaster* (third instar larval wing disc), *Xenopus laevis* (tadpole tail epidermis), *Hydra vulgaris* (adult outer epidermis) comes from our previous work [3]. The data for Anagallis arvensis (meristem) was given to us courtesy of Jacques Dumais and derived from Figure 1 in [4]. The cucumber epidermis data was taken from F.T. Lewis' original papers [5],[6]. Modes are shown in red.
(0.04 MB DOC)

Table S2 Cell shape distribution data for all CPMs. Distribution data for simulated CPMs. Each data point is a result of 100 simulations, each with 12 generations of division and 4,096 cells. Modes of distributions are shown in red. This data supports the existence of an equilibrium distribution that depends on the CPM but is independent of initial conditions.
(0.07 MB DOC)

Table S3 Cell shape distribution data for all CPMS (Sorted by percentage of hexagons). The same shape distribution data for simulated CPMs as shown in Table 2 but here sorted by the steady state fraction of hexagonal cells. As in Table 2, each data point is a result of 100 simulations, each with 12 generations of division and 4,096 cells. Modes of distributions are shown in red. Hexagonal frequencies are shown in bold. As division becomes more symmetric and charitable, the fraction of hexagons increases and eventually hexagons become the mode of the shape distribution.
(0.08 MB DOC)

Table S4 Standard Deviation (%) of Equilibrium Fraction of *n*-sided cells. Standard deviation of distribution data for simulated CPMs. To test convergence, each simulated CPM was run on several initial conditions with 100 independent runs each to calculate the equilibrium cell shape distributions shown in Tables S2 and S3. This table shows the standard deviation for each cell shape category across different runs. Almost all simulations show less than 1% deviation in cell shape percentages, indicating that each division rule (CPM) generates a robust signature distribution with little variability. Large standard deviations (exceeding 1.0%) are shown in blue. Rules with maximally uncharitable division strategies (LARGESTNEIGHBOR_1_ Side_1_ strategy) appear more likely to exhibit high variability in equilibrium shape frequency. The large variance appears to be a result of conflicting effects that increase and decrease shape variance (e.g. LARGESTNEIGHBOR|EQUALSPLIT) causing the overall topology to be unstable. In contrast LARGESTNEIGHBOR|UNEQUALSPLIT quickly converges to a stable situation with 99.9% quadrilaterals and one extremely large cell.
(0.07 MB DOC)

Table S5 Comparison to other Relevant Models.
(0.03 MB DOC)

Text S1 Includes relevant data, methodologies, and equations that supplement the main manuscript.
(0.05 MB DOC)
