# Supplementary figures and images for: Modeling and Inferring Cleavage Patterns in Proliferating Epithelia
Source: PLoS Comput Biol. 2009 Jun 12;5(6):e1000412. doi: 10.1371/journal.pcbi.1000412 (PMC2688032; doi:10.1371/journal.pcbi.1000412)

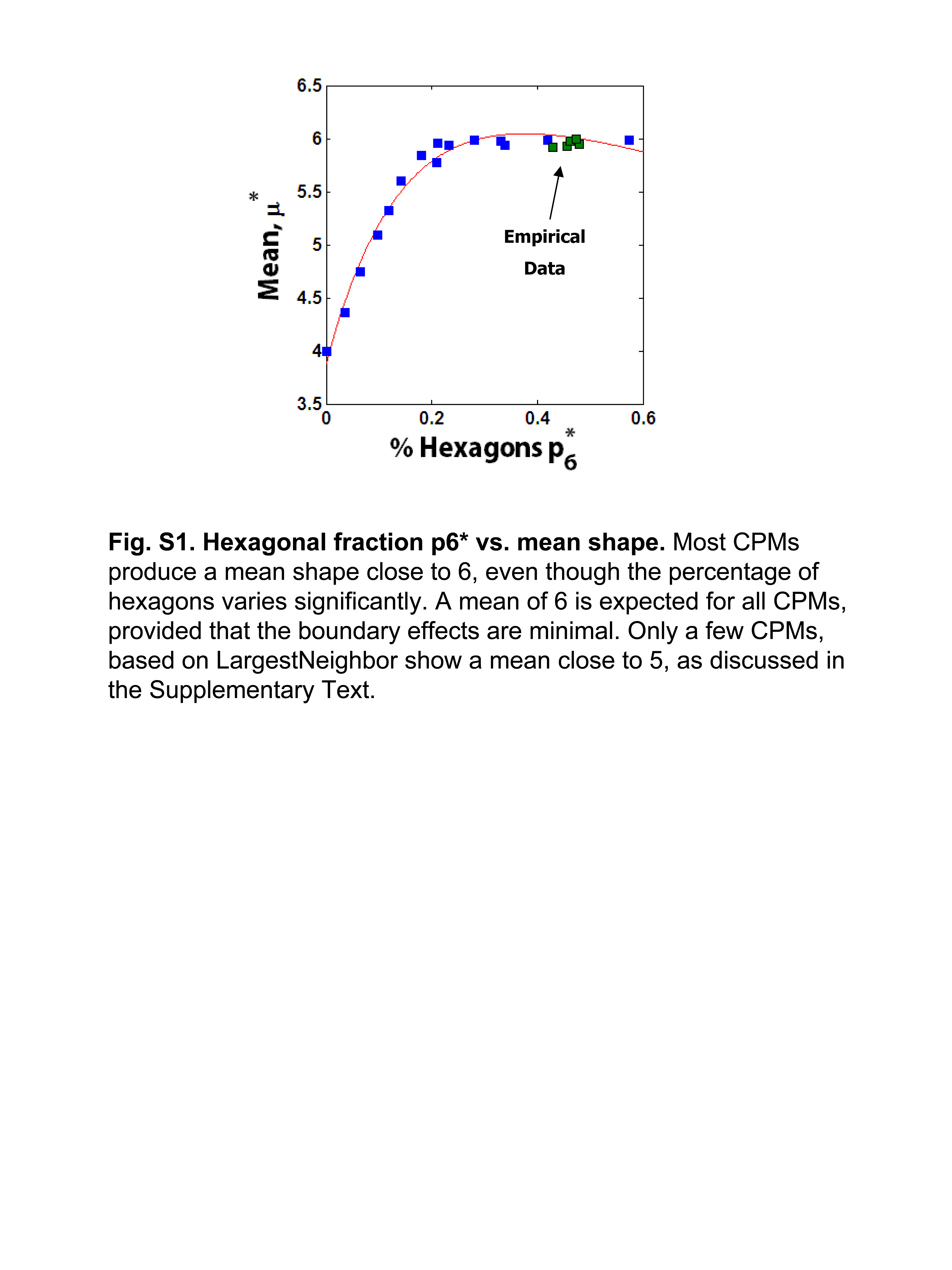

Supplement: Figure S1 — Hexagonal fraction p6* vs. mean shape. Most CPMs produce a mean shape close to 6, even though the percentage of hexagons varies significantly. A mean of 6 is expected for all CPMs, provided that the boundary effects are minimal. Only a few CPMs, based on LargestNeighbor1 show a mean closer to 5, as discussed in the Supplementary text. (3.46 MB TIF) [file pcbi.1000412.s001.tif]

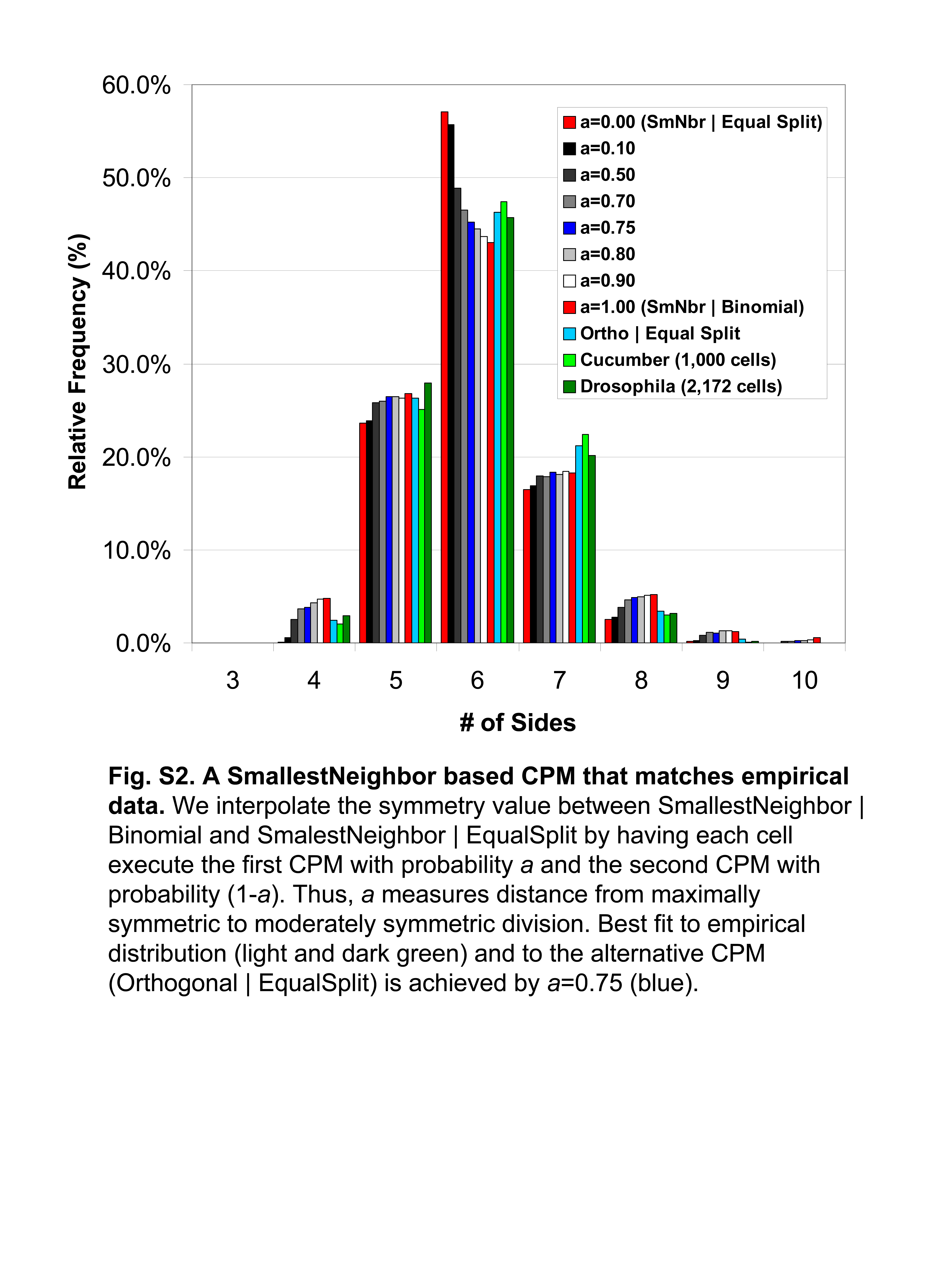

Supplement: Figure S2 — A SmallestNeighbor based CPM that matches empirical data. We interpolate the symmetry value between SmallestNeighbor|Binomial and SmallestNeighbor|EqualSplit by having each cell execute the first method with probability a and the second method with probability (1-a). Thus, a measures distance from maximally symmetric to moderately symmetric division. Best fit to empirical distribution (light and dark green) and to the alternative CPM (Orthogonal|EqualSplit) is achieved by a = 0.75 (blue). (5.22 MB TIF) [file pcbi.1000412.s002.tif]
