# Supplementary material for: Modeling and Inferring Cleavage Patterns in Proliferating Epithelia
Source: PLoS Comput Biol. 2009 Jun 12;5(6):e1000412. doi: 10.1371/journal.pcbi.1000412 (PMC2688032; doi:10.1371/journal.pcbi.1000412)
Supplement: Table S1 — Empirical Cell Shape Distribution Data from Five Diverse Organisms. Shape distribution data for proliferating epithelial in several organisms of interest. The data for Drosophila melanogaster (third instar larval wing disc), Xenopus laevis (tadpole tail epidermis), Hydra vulgaris (adult outer epidermis) comes from our previous work [3]. The data for Anagallis arvensis (meristem) was given to us courtesy of Jacques Dumais and derived from Figure 1 in [4]. The cucumber epidermis data was taken from F.T. Lewis' original papers [5],[6]. Modes are shown in red. (0.04 MB DOC) [file pcbi.1000412.s003.doc]

**SI Table 1. Empirical Cell Shape Distribution Data from Five Diverse Organisms**

|  | | ***n*** | | | |  |  |  |
| --- | --- | --- | --- | --- | --- | --- | --- | --- |
| ***Organism*** | 3 | | 4 | 5 | 6 | 7 | 8 | 9 |
| Drosophila (2,172 cells) |  | | 2.95% | 27.90% | 45.72% | 20.12% | 3.18% | 0.14% |
| Xenopus (1,051 cells) | 0.19% | | 3.81% | 29.02% | 42.91% | 18.17% | 4.95% | 0.76% |
| Hydra (602 cells) |  | | 2.66% | 26.41% | 46.18% | 20.76% | 3.82% | 0.17% |
| Anagallis (392 cells) |  | | 2.81% | 26.53% | 47.96% | 18.88% | 3.83% | 0.00% |
| Cucumber (1,000 cells) |  | | 2.00% | 25.10% | 47.40% | 22.40% | 3.00% | 0.10% |
| Cucumber Mitotic cells (1,000 cells) | | | 0.00% | 1.60% | 25.50% | 47.80% | 22.40% | 2.60% |

Shape distribution data for proliferating epithelial in several organisms of interest. The data for *Drosophila melanogaster (third instar larval wing disc)*, *Xenopus laevis (tadpole tail epidermis)*, *Hydra vulgaris* (*adult outer epidermis)* comes from our previous work (3). The data for *Anagallis arvensis* *(meristem)* was given to us courtesy of Jacques Dumais and derived from Fig. 1 in (4). The cucumber epidermis data was taken from F.T. Lewis’ original papers (5,6). Modes are shown in **red**.
