# Supplementary material for: Modeling and Inferring Cleavage Patterns in Proliferating Epithelia
Source: PLoS Comput Biol. 2009 Jun 12;5(6):e1000412. doi: 10.1371/journal.pcbi.1000412 (PMC2688032; doi:10.1371/journal.pcbi.1000412)
Supplement: Table S2 — Cell shape distribution data for all CPMs. Distribution data for simulated CPMs. Each data point is a result of 100 simulations, each with 12 generations of division and 4,096 cells. Modes of distributions are shown in red. This data supports the existence of an equilibrium distribution that depends on the CPM but is independent of initial conditions. (0.07 MB DOC) [file pcbi.1000412.s004.doc]

**SI Table 2. Cell shape distribution data for all CPMS**

|  | | | | |  |  | ***n*** |  |  |  |  |  |
| --- | --- | --- | --- | --- | --- | --- | --- | --- | --- | --- | --- | --- |
| **CPM (Side1 | Side2)** |  | | **3** | **4** | **5** | **6** | **7** | **8** | **9** | **10** | **11** | **12** |
| Random | Random | | 0.00% | | 26.98% | 26.86% | 18.08% | 11.00% | 6.56% | 3.90% | 2.43% | 1.46% | 0.94% |
| Random | Binomial | | 0.00% | | 18.43% | 27.67% | 23.34% | 15.06% | 8.26% | 4.06% | 1.87% | 0.80% | 0.32% |
| Random | EqualSplit | | 0.00% | | 8.04% | 29.87% | 33.80% | 19.19% | 6.83% | 1.84% | 0.35% | 0.06% | 0.01% |
| Random | UnequalSplit | | 0.00% | | 42.58% | 23.82% | 11.88% | 6.44% | 4.11% | 2.69% | 1.88% | 1.38% | 0.95% |
| Orthogonal | Random | | 0.00% | | 19.85% | 29.21% | 21.15% | 13.06% | 7.54% | 4.12% | 2.24% | 1.25% | 0.71% |
| Orthogonal | Binomial | | 0.00% | | 11.90% | 29.19% | 28.14% | 17.46% | 8.42% | 3.27% | 1.14% | 0.35% | 0.10% |
| Orthogonal | EqualSplit | | 0.00% | | 2.36% | 26.25% | 46.53% | 21.03% | 3.55% | 0.27% | 0.01% | 0.00% | 0.00% |
| Orthogonal | UnequalSplit | | 0.00% | | 34.99% | 25.70% | 14.27% | 7.98% | 5.07% | 3.28% | 2.23% | 1.58% | 1.13% |
| SmallestNbr | Random | | 0.00% | | 9.99% | 29.82% | 33.10% | 15.51% | 6.57% | 2.78% | 1.20% | 0.54% | 0.28% |
| SmallestNbr | Binomial | | 0.00% | | 5.27% | 27.06% | 41.88% | 18.24% | 5.57% | 1.50% | 0.35% | 0.09% | 0.02% |
| SmallestNbr | EqualSplit | | 0.00% | | 0.08% | 23.45% | 57.38% | 16.39% | 2.45% | 0.22% | 0.02% | 0.00% | 0.00% |
| SmallestNbr | UnequalSplit | | 0.00% | | 21.54% | 32.41% | 20.92% | 10.29% | 5.51% | 3.04% | 1.91% | 1.23% | 0.83% |
| LargestNbr | Random | | 0.00% | | 78.02% | 10.23% | 3.50% | 1.76% | 1.06% | 0.76% | 0.54% | 0.42% | 0.33% |
| LargestNbr | Binomial | | 0.00% | | 60.35% | 18.55% | 6.56% | 3.13% | 1.95% | 1.36% | 1.04% | 0.82% | 0.70% |
| LargestNbr | EqualSplit | | 0.00% | | 46.65% | 27.01% | 9.77% | 3.96% | 2.21% | 1.64% | 1.30% | 1.15% | 0.94% |

Distribution data for simulated CPMs. Each data point is a result of 100 simulations, each with 12 generations of division and 4,096 cells. Modes of distributions are shown in **red**. This data supports the existence of an equilibrium distribution that depends on the CPM but is independent of initial conditions.
