# Supplementary material for: Modeling and Inferring Cleavage Patterns in Proliferating Epithelia
Source: PLoS Comput Biol. 2009 Jun 12;5(6):e1000412. doi: 10.1371/journal.pcbi.1000412 (PMC2688032; doi:10.1371/journal.pcbi.1000412)
Supplement: Table S3 — Cell shape distribution data for all CPMS (Sorted by percentage of hexagons). The same shape distribution data for simulated CPMs as shown in Table 2 but here sorted by the steady state fraction of hexagonal cells. As in Table 2, each data point is a result of 100 simulations, each with 12 generations of division and 4,096 cells. Modes of distributions are shown in red. Hexagonal frequencies are shown in bold. As division becomes more symmetric and charitable, the fraction of hexagons increases and eventually hexagons become the mode of the shape distribution. (0.08 MB DOC) [file pcbi.1000412.s005.doc]

**SI Table 3. Cell shape distribution data for all CPMS (Sorted by percentage of hexagons)**

|  |  |  |  |  |  |  |  |  |  |  |
| --- | --- | --- | --- | --- | --- | --- | --- | --- | --- | --- |
|  |  |  |  | ***n*** |  |  |  |  |  |  |
| **CPM (Side1|Side2)** | **3** | **4** | **5** | **6** | **7** | **8** | **9** | **10** | **11** | **12** |
| SmallestNbr | EqualSplit | 0.00% | 0.08% | 23.45% | **57.38%** | 16.39% | 2.45% | 0.22% | 0.02% | 0.00% | 0.00% |
| Orthogonal | EqualSplit | 0.00% | 2.36% | 26.25% | **46.53%** | 21.03% | 3.55% | 0.27% | 0.01% | 0.00% | 0.00% |
| SmallestNbr | Binomial | 0.00% | 5.27% | 27.06% | **41.88%** | 18.24% | 5.57% | 1.50% | 0.35% | 0.09% | 0.02% |
| Random | EqualSplit | 0.00% | 8.04% | 29.87% | **33.80%** | 19.19% | 6.83% | 1.84% | 0.35% | 0.06% | 0.01% |
| SmallestNbr | Random | 0.00% | 9.99% | 29.82% | **33.10%** | 15.51% | 6.57% | 2.78% | 1.20% | 0.54% | 0.28% |
| Orthogonal | Binomial | 0.00% | 11.90% | 29.19% | **28.14%** | 17.46% | 8.42% | 3.27% | 1.14% | 0.35% | 0.10% |
| Random | Binomial | 0.00% | 18.43% | 27.67% | **23.34%** | 15.06% | 8.26% | 4.06% | 1.87% | 0.80% | 0.32% |
| Orthogonal | Random | 0.00% | 19.85% | 29.21% | **21.15%** | 13.06% | 7.54% | 4.12% | 2.24% | 1.25% | 0.71% |
| SmallestNbr | UnequalSplit | 0.00% | 21.54% | 32.41% | **20.92%** | 10.29% | 5.51% | 3.04% | 1.91% | 1.23% | 0.83% |
| Random | Random | 0.00% | 26.98% | 26.86% | **18.08%** | 11.00% | 6.56% | 3.90% | 2.43% | 1.46% | 0.94% |
| Orthogonal | UnequalSplit | 0.00% | 34.99% | 25.70% | **14.27%** | 7.98% | 5.07% | 3.28% | 2.23% | 1.58% | 1.13% |
| Random | UnequalSplit | 0.00% | 42.58% | 23.82% | **11.88%** | 6.44% | 4.11% | 2.69% | 1.88% | 1.38% | 0.95% |
| LargestNbr | EqualSplit | 0.00% | 46.65% | 27.01% | **9.77%** | 3.96% | 2.21% | 1.64% | 1.30% | 1.15% | 0.94% |
| LargestNbr | Binomial | 0.00% | 60.35% | 18.55% | **6.56%** | 3.13% | 1.95% | 1.36% | 1.04% | 0.82% | 0.70% |
| LargestNbr | Random | 0.00% | 78.02% | 10.23% | **3.50%** | 1.76% | 1.06% | 0.76% | 0.54% | 0.42% | 0.33% |
| LargestNbr | UnequalSplit | 0.00% | 99.30% | 0.06% | **0.04%** | 0.03% | 0.03% | 0.02% | 0.02% | 0.02% | 0.01% |

The same shape distribution data for simulated CPMs as shown in SI Table 2 but here sorted by the steady state fraction of hexagonal cells. As in SI Table 2, each data point is a result of 100 simulations, each with 12 generations of division and 4,096 cells. Modes of distributions are shown in **red**. Hexagonal frequencies are shown in **bold**. As division becomes more symmetric and charitable, the fraction of hexagons increases and eventually hexagons become the mode of the shape distribution.
