# Supplementary material for: Modeling and Inferring Cleavage Patterns in Proliferating Epithelia
Source: PLoS Comput Biol. 2009 Jun 12;5(6):e1000412. doi: 10.1371/journal.pcbi.1000412 (PMC2688032; doi:10.1371/journal.pcbi.1000412)
Supplement: Table S4 — Standard Deviation (%) of Equilibrium Fraction of n-sided cells. Standard deviation of distribution data for simulated CPMs. To test convergence, each simulated CPM was run on several initial conditions with 100 independent runs each to calculate the equilibrium cell shape distributions shown in Tables S2 and S3. This table shows the standard deviation for each cell shape category across different runs. Almost all simulations show less than 1% deviation in cell shape percentages, indicating that each division rule (CPM) generates a robust signature distribution with little variability. Large standard deviations (exceeding 1.0%) are shown in blue. Rules with maximally uncharitable division strategies (LARGESTNEIGHBOR1 Side1 strategy) appear more likely to exhibit high variability in equilibrium shape frequency. The large variance appears to be a result of conflicting effects that increase and decrease shape variance (e.g. LARGESTNEIGHBOR|EQUALSPLIT) causing the overall topology to be unstable. In contrast LARGESTNEIGHBOR|UNEQUALSPLIT quickly converges to a stable situation with 99.9% quadrilaterals and one extremely large cell. (0.07 MB DOC) [file pcbi.1000412.s006.doc]

**SI Table 4. Standard Deviation (%) of Equilibrium Fraction of *n*-sided cells *p_n_****

|  | | | | |  |  |  |  |  |  |
| --- | --- | --- | --- | --- | --- | --- | --- | --- | --- | --- |
| **CPM (Side1 | Side2)** | **3** | **4** | **5** | **6** | **7** | **8** | **9** | **10** | **11** | **12** |
| Random | Random | 0.0% | 0.6% | 0.8% | 0.5% | 0.5% | 0.4% | 0.3% | 0.3% | 0.2% | 0.2% |
| Random | Binomial | 0.0% | 0.6% | 0.6% | 0.7% | 0.5% | 0.4% | 0.3% | 0.2% | 0.1% | 0.1% |
| Random | EqualSplit | 0.0% | 0.5% | 0.7% | 0.7% | 0.5% | 0.4% | 0.2% | 0.1% | 0.0% | 0.0% |
| Random | UnequalSplit | 0.0% | 0.5% | 0.6% | 0.4% | 0.4% | 0.3% | 0.2% | 0.2% | 0.2% | 0.2% |
| Orthogonal | Random | 0.0% | 0.5% | 0.7% | 0.5% | 0.5% | 0.4% | 0.3% | 0.2% | 0.2% | 0.1% |
| Orthogonal | Binomial | 0.0% | 0.4% | 0.7% | 0.6% | 0.6% | 0.4% | 0.2% | 0.2% | 0.1% | 0.0% |
| Orthogonal | EqualSplit | 0.0% | 0.2% | 0.5% | 0.8% | 0.6% | 0.3% | 0.1% | 0.0% | 0.0% | 0.0% |
| Orthogonal | UnequalSplit | 0.0% | 0.4% | 0.6% | 0.5% | 0.4% | 0.3% | 0.3% | 0.2% | 0.2% | 0.2% |
| SmallestNbr | Random | 0.0% | 0.5% | 0.8% | 0.7% | 0.5% | 0.3% | 0.3% | 0.2% | 0.1% | 0.1% |
| SmallestNbr | Binomial | 0.0% | 0.3% | 0.6% | 0.6% | 0.5% | 0.3% | 0.2% | 0.1% | 0.1% | 0.0% |
| SmallestNbr | EqualSplit | 0.0% | 0.0% | 0.5% | 0.8% | 0.5% | 0.2% | 0.1% | 0.0% | 0.0% | 0.0% |
| SmallestNbr | UnequalSplit | 0.0% | 0.3% | 0.4% | 0.5% | 0.4% | 0.3% | 0.2% | 0.2% | 0.2% | 0.1% |
| LargestNbr | Random | 0.0% | 3.4% | 1.6% | 0.6% | 0.4% | 0.2% | 0.2% | 0.1% | 0.1% | 0.1% |
| LargestNbr | Binomial | 0.0% | 5.4% | 2.4% | 1.2% | 0.7% | 0.4% | 0.3% | 0.2% | 0.2% | 0.2% |
| LargestNbr | EqualSplit | 0.0% | 5.7% | 2.6% | 1.4% | 0.7% | 0.5% | 0.4% | 0.4% | 0.3% | 0.2% |
| LargestNbr | UnequalSplit | 0.0% | 0.1% | 0.1% | 0.0% | 0.0% | 0.0% | 0.0% | 0.0% | 0.0% | 0.0% |

Standard deviation of distribution data for simulated CPMs. To test convergence, each simulated CPM was run on several initial conditions with 100 independent runs each to calculate the equilibrium cell shape distributions shown in Tables S2 and S3. This table shows the standard deviation for each cell shape category across different runs. Almost all simulations show less than 1% deviation in cell shape percentages, indicating that each division rule (CPM) generates a robust signature distribution with little variability. Large standard deviations (exceeding 1.0%) are shown in **blue**. Rules with maximally uncharitable division strategies (LargestNeighbor_1_ Side1 strategy) appear more likely to exhibit high variability in equilibrium shape frequency. The large variance appears to be a result of conflicting effects that increase and decrease shape variance (e.g. LargestNeighbor | Equalsplit) causing the overall topology to be unstable. In contrast LargestNeighbor | Unequalsplit quickly converges to a stable situation with 99.9% quadrilaterals and one extremely large cell.
