# Supplementary material for: Modeling and Inferring Cleavage Patterns in Proliferating Epithelia
Source: PLoS Comput Biol. 2009 Jun 12;5(6):e1000412. doi: 10.1371/journal.pcbi.1000412 (PMC2688032; doi:10.1371/journal.pcbi.1000412)
Supplement: Table S5 — Comparison to other Relevant Models. (0.03 MB DOC) [file pcbi.1000412.s007.doc]

**SI Table 5. Comparison to other Relevant Models**

| ***Aspects of Model*** | ***Current topological simulation model*** | ***Dubertret et al.*** | ***Faarhadifar et al*** | ***Markov model*** |
| --- | --- | --- | --- | --- |
| **Epithelial representations** | Topological | Topological | Geometrical/Mechanical | Topological, Markov |
| **Simulation Modes** | Division only | Division and/or Disappearance | Division and/or Disappearance | Division only |
| **Notion of Time** | generations | # of times a cell was randomly chosen for division | generations | generations |
| **Division probability** | all cells divide | Shape-dependent | all cells divide | all cells divide |
| **Side1** | arbitrary | Random | Random (geometric) | unspecified |
| **Side2** | arbitrary | EqualSplit | Cleavage plane is a randomly chosen line segment that passes through the cell centroid | Binomial |
| **Initial conditions** | 1 hexagonal cell, | 10,000 hexagonal cells with optional 300,000 random T1 transformations (exchanges) | 36 hexagonal cells | arbitrary |
| **Boundary conditions** | N/A | periodic | periodic | N/A |
