# Supplementary material for: Modeling and Inferring Cleavage Patterns in Proliferating Epithelia
Source: PLoS Comput Biol. 2009 Jun 12;5(6):e1000412. doi: 10.1371/journal.pcbi.1000412 (PMC2688032; doi:10.1371/journal.pcbi.1000412)
Supplement: Text S1 — Includes relevant data, methodologies, and equations that supplement the main manuscript. (0.05 MB DOC) [file pcbi.1000412.s008.doc]

**Text S1**

**Equations and Parameters for Simulated CPMs**

Here we give the mathematical formulas underlying the Side1 and Side2 models tested in this paper. Side1 models choose the first edge of a cleavage plane. Let *q_A_(k)* denote an *n*-sided cell’s probability of choosing edge *k* as Side1 under CPM A. Let *n_k_* be the number of sides of neighbor *k*. In the formulas below, *q_A_(k)* = 0 if it is not defined for some *k*. Then a cell chooses Side1 according to one of the following equations:

Now let *l* be in the set {0, 1, …, *n-*1} denote the *l*th clockwise edge on the periphery of an *n*-sided cell and without loss of generality, let *l* = 0 denote the Side1 edge already chosen by the Side1 model above. For instance, *l* = 1 and *l* = *n-*1 are the two edges to the left and right of Side1. Let *r_A_(l)* denote the probability of choosing edge *l* as Side 2 under CPM A. In the formulas below, *r_A_(l)* = 0 if it is not defined for some *l*.

Results of simulations are shown in Fig. 3 and in Tables S2-4.

Simulations were run for 12 generations (with random ordering of cell divisions within each generation). The choice of number of generations was motivated by proliferation in the larval fruit fly wing and by a previous Markov model (3) which indicated that 6-8 rounds were sufficient for convergence. Most simulations converged within 8 rounds, with minimal variation between cell shape distributions observed in different runs (Table S4).

**Empirical Distribution Data**

Shape data was hand-counted from each of the following five diverse organisms: *Drosophila melanogaster (third instar larval wing)*, *Xenopus laevis (tadpole tail epidermis)*, *Hydra vulgaris* (*outer epidermis), Anagallis arvensis* *(meristem), and cucumber (epidermis).* For additional information on the first three data sets, see reference (3). The *Anagallis* data was a kind gift from Jacques Dumais (see Fig. 1 in ref. 4) and all cucumber data is from F.T. Lewis’s original papers (5, 6). See Table S1 for a summary of the different shape frequencies. Our data on *Drosophila* larval wing differs from that of Farhadifar *et al.* (2), most likely due to the method and resolution of staining septate junctions that allows us to resolve small edges which may otherwise be scored as 4-junctions. We also compared our hand counts to those automatically extracted by *Definiens* software; the resulting counts are close, confirming the accuracy of our hand counts.

**Distribution Data from Simulated CPMs**

See Tables S2-4. For comparison to empirical data, see Figure 3A and Table S1.

**Boundary effects in computing the mean shape**

Previously we showed that the mean shape of an epithelium should converge quickly to hexagonal (3). This result depends on boundary affects being small, which in turn depends on roughly isotropic expansion of clones. For example, consider a rectangular cell that divides in the north-south direction into two daughter cells; if each daughter repeats a parallel division then the result is a long strip of rectangular cells with a mean cell shape of 4, not 6. This is a result of the fact that most junctions are being created along the tissue boundary. In our previous work, we described mathematically how to derive the mean including such boundary effects (3, supplemental text).

Most of our models do not display significant boundary effects, and the mean is close to 6. For example, SmallestNbr|EqualSplit and Orthogonal|EqualSplit both have a mean cell shape of 5.98. However, the mathematically-motivated CPMs where a cell divides in the direction of the largest neighbor show low mean cell shape, e.g. LargestNbr|UnequalSplit generates a steady state mean of 4.83. The final topology of this CPM provides an indication of how this occurs: this division method favors a few large cells that are surrounded by a boundary ring of small cells and the boundary ring constantly expands as small cells favor dividing in the direction of the middle cell. This type of topology is biologically implausible but demonstrates how a regular division mechanism can produce a highly irregular equilibrium distribution.

**Comparison to Other Models**

*Dubertret et al. model (1)*

Dubertret *et al.* developed a topological simulation model for studying the effect of cell proliferation and cell death. The work presented here differs from the topological simulation model of Dubertret *et al.* in two important respects: (a) the cleavage plane division model, and (b) the timing model.

*CPM Model:* One major difference between our approach and that of Dubertret *et al.* is the choice of CPM. Dubertret *et al.* focus on a single model of cleavage plane choice where cells divide into equal daughters without any influence from neighboring cells. Their model is equivalent to a Random | EqualSplit CPM. Instead of exploring different CPMs, their model incorporates other aspects such as cell rearrangement (also known as T1 transformations), cell death and different probabilities of division. At each step, a cell is chosen and with some probability the cell may divide, rerrange or disappear. They explore a large space of tunable parameters and functions. One difficulty with this model is that while they are able to reproduce Lewis’s distribution data in the cucumber epidermis, the model accepts 18 tunable parameters making it difficult to extract any mechanistic understanding (model “h” in (1)). Our work instead focuses on CPM models, specifically the influence of division symmetry and neighborhoods, and does not model cell rearrangement or cell death. Our work shows that the CPM model can have a strong impact on the outcome of proliferation, and that local interactions between a cell and its neighborhood can significantly change the global equilibrium topology’s degree distribution. In future work, as the model is tailored for different organisms, it may be possible to add in more complexity while using experimentally-derived parameters to constrain the space of models.

*Timing model:* A second significant difference is the timing model with which cells divide. In their topological simulations, the system begins with a sheet of 10,000 hexagonal cells and at each step a cell is randomly chosen to divide. In contrast, our timing model assumes that cells divide exactly once in a random order for each of 12 rounds. These two models (random timing and round-based) represent two extremes. In the round-based model, due to the random ordering, a cell can divide twice before its neighbor, but eventually all cells will divide an equal number of times. In the random timing model, the number of divisions a cell undergoes has large variability across the sheet; some cells may not divide at all while others may divide multiple times.

Our round-based model is motivated by data from the *Drosophila* larval wing disc. In the wing disc it has been experimentally established that there is a relatively uniform rate of cell cycle progression throughout the tissue (7). Cells are thought to divide according to an approximate 8-12 hour cell cycle, with faster cycles early on, and slower cycles towards the end of larval development. This would make it highly unlikely that any one cell could divide twice in a period of time where its neighbors do not divide once. In addition, cells that divide too slowly are subject to a process known as 'cell competition' where slow dividing cells are culled from the population through apoptotic cell death. For these reasons, the timing model which simulates rounds of division is more appropriate, if imperfect.

Nevertheless, different timing models can have a significant effect on the cell shape distribution. We have simulated the effect of Dubertret’s random timing model on two CPMs: SmallestNbr|Binomial and Ortho|EqualSplit . In these simulations, the system starts with a single hexagonal cell, and at each step a cell is chosen uniformly at random to divide, until the topology contains 4000 cells. The resulting distributions are:

SmallestNbr|Binomial

[15.6%(4) 31.4%(5) 24.1%(6) 13.8%(7) 7.3%(8) 3.7%(9) 1.9%(10) 1.0%(11)]

Ortho|EqualSplit

[14.2%(4) 29.1%(5) 28.7%(6) 14.6%(7) 6.7% (8) 3.2%(9) 1.6%(10) 0.8%(11)]

The random timing model shows a significantly lower percentage of hexagonal cells and a higher percentage of cells with 9, 10, and even 11 sides. It also fails to reproduce a second aspect of the data, called the mitotic shift. In both *Drosophila* and cucumber it has been observed that the distribution of mitotic cells shows a mean of 7 (shifted by 1 from the normal cell distribution) (3,5,6). From the simulation, we can compute the mitotic shift as the mean shape over all dividing (mitotic) cells. In the random timing model, neither CPM produces a mitotic shift (mitotic mean=5.84 and 5.85 respectively). In contrast, the round-based timing model does produce a mitotic shift for both CPMs (mitotic mean=6.93 and 6.70). The intuition is that cells chosen for division later in the cycle are more likely to have a greater number of sides simply because their neighbors have already divided. In fact, this logic holds for all simulated CPMs: the mean shape for mitotic cells was always significantly greater than six, although the exact value was CPM-dependent, ranging from 6.69 - 6.94 sides.

While the random timing model may not be appropriate for modeling proliferating epithelial tissues, there are other potential timing models, such as a shape-dependent stochastic division. Dubertret *et al.* explore a range of parameterized functions beyond the random timing model, but do not consider round-based models. Further exploration of the division timing model, both theoretically and experimentally in the *Drosophila*, will be an important area of future research.

In spite of these differences, both our work and that of Dubertret *et al.* show how local cell decisions can impact final topology, and both show that certain types of cell decisions can suppress shape variability in the epithelium, forcing very large and very small cells to either divide or disappear, thus bringing the epithelium closer to the mean hexagonal shape.

*Farhadifar et al. model (2)*

Farhadifar *et al* developed a mechanical model of cell proliferation, where a cell is represented by a combination of physical forces, such as surface-area/perimeter conservation and tension between neighboring cells. The simulation uses an energy function and a relaxation model to compute the tissue geometry after each cell division. The model requires several parameters for defining the energy function, and uses Lewis’s Law of cell areas to partially constrain these parameters. In addition, the model allows for cellular repacking (i.e. neighbor exchanges) directed by physical forces trying to minimize tensional energy in the epithelium. By contrast, our model uses a topological representation and assumes that there is no repacking, consistent with marked clone data and time-lapse movies of proliferation in the *Drosophila* wing disc (3).

Although the model of Farhadifar *et al* is geometric rather than topological, it is still possible to make a comparison with their results. While their model is able to reproduce Lewis’ Law for average cell areas, the same parameters that achieve this also generate a polygonal cell shape distribution quite different from that seen empirically. For instance, the shape distribution has a mode of 5 (pentagons), whereas the empirical distribution has a mode of 6 (hexagons). In our framework, the model lies somewhere along the spectrum between Random | EqualSplit (mode=6) and Random | Binomial (mode=5). To achieve a mode of 6 (hexagons), our models suggest that local cleavage must be either highly symmetric (e.g. * | EqualSplit) and/or highly charitable (e.g. SmallestNeighbor | *). See Table S3 for all CPMs that generate modes of 5 and 6. Together, this evidence indicates there are still key unknown factors that control cell division in the *Drosophila* wing disc. A next step is to formulate a geometrical model that reproduces both the topological statistics (e.g. the cell shape distribution) and the geometrical statistics (e.g. average and variance in cell areas) observed empirically in proliferating epithelia.

*Our previous model (3)*

In our previous work (3) we developed a mathematical Markov chain model to describe proliferation in the Drosophila larval wing disc. This model made two main predictions that motivate the current work: (a) proliferation produces an equilibrium distribution, that is independent of initial state but dependant on division symmetry and (b) that a specific division model predicts the distribution seen in the proliferating larval wing disc as well as several other epithelia. However the model suffers from some limitations, in particular neighbor effects are modeled using a global mean-field approximation. This simplification facilitates mathematical analysis but does not deal with the complexities associated with neighbor correlations.

Our topological simulations shed light on both predictions of the previous model. First, the simulations verify that a large spectrum of more complex division models *qualitatively* exhibit the same behavior as the original model, i.e. convergence to an equilibrium cell shape distribution, that is independent of initial state but reflects division parameters. Proving that the simulation models converge remains an open and difficult mathematical problem.

Second, the topological simulations show that the previous model did not completely capture the requirements for producing the *Drosophila* wing disc cell shape distributions. The previously posited mechanism can be thought of as *|Binomial CPM where * is unspecified and approximated as having a mean-field effect where all cells gain one side in each division generation. This does not distinguish between different algorithms for the choice of Side1. A naive translation of this mathematical model into the Random|Binomial CPM does not generate *p**. Indeed, the distribution generated has a significantly larger variance than that observed empirically (see Table S2). However, the SmallestNeighbor|Binomial CPM does generate a shape distribution that is similar to that observed empirically, suggesting that high charitability is necessary to produce the effect assumed by the mean-field approximation.

Our work suggests that using a single global mean-field approximation is not a good technique for modeling epithelial proliferation, since the neighbor effects appear to have significant impact. However this still leaves open the question whether appropriate Markov models can be created to capture and predict the simulation data, and whether one can prove convergence without using Markovian approximations. We have explored the ability to create data-derived Markov models from topological simulations, and this shows some promise (chapter 4, (8)). However, this remains an active area of research.

**References**

1. Dubertret B, Aste T, Ohlenbusch H, Rivier N (1998) Two-Dimensional froths and the dynamics of biological tissues. *Phys Rev E* 58:6368-6378.
2. Farhadifar R, Röper JC, Aigouy B, Eaton S, Jülicher F (2007) The Influence of Cell Mechanics, Cell-Cell Interactions, and Proliferation on Epithelial Packing. *Current Biology* 17:2095–2104.
3. Gibson MG, Patel AB, Nagpal R, Perrimon N (2006) Emergence of geometric order in proliferating epithelia. *Nature* 442:1038-1041.
4. Kwiatkowska D, Dumais J (2003) Growth and morphogenesis at the vegetative shoot apex of Anagallis arvensis. *Journal of Experimental Botany* 54:1585-1595.
5. Lewis F-T (1926) The effect of cell division on the shape and size of hexagonal cells. *Anat Rec* 33:331-355.
6. Lewis F-T (1928) The correlation between cell division and the shapes and sizes of prismatic cells in the epidermis of Cucumis. *Anat Rec* 38:341-376.
7. Milan M, Campuzano S, Garcia-Bellido A (1996) Cell cycling and patterned cell proliferation in the wing primordium of Drosophila. Proc. Natl Acad. Sci. USA 93, 640–-645.
8. Patel A (2008) Modeling and Inferring Cleavage Patterns in Proliferating Epithelia. Doctoral Thesis (Applied Mathematics), Harvard University.
